# Remarkable Effect upon the Human Gums, Produced by Absorption of Lead

**Published:** 1841-09

**Authors:** Henry Burton

**Affiliations:** physician to St. Thomas' hospital.


### ARTCLE IX.

*Remarkable effect upon the Human Gums, produced by absorption of Lead.* By HENRY BURTON, M. D. physician to St. Thomas' hospital.

THE only approach to the observation now to be stated, is a notice by Dr. Christison, and Dr. A. T. Thomson, that in persons who have taken lead in quantity, salivation is apt to occur, and the saliva is of a *bluish colour*. Dr. Burton, from observations made since 1834, on persons who had been exposed to the action of lead in the course of their usual avocations, and of those also who had swallowed the acetate of lead medicinally, is of opinion, that salivation in the ordinary sense of the term, does not occur in one case out of thirty-six cases of lead colic, the number examined in his wards; nor in one case out of fourteen cases of pulmonary disease, which were treated by him with acetate of lead, but in the total number of fifty patients, who were examined whilst under the influence of lead, a peculiar discoloration was observed on their gums, which he could not discern on the gums of several hundred patients who were not under its influence.

The edges of the gums attached to the necks of two or more teeth of either jaw, were distinctly bordered by a *narrow leaden-blue* line, about the twentieth of an inch in width, whilst the substance apparently retained its ordinary colour and condition. There was no invariable tumefaction, softening, or tenderness about them—nor fœtor of the breath—while the quantity and colour of the saliva preserved the same appearance after as before the appearance of the blue line.

This appearance has no resemblance to the effects caused by mercury. On the contrary, the blue line was obliterated on patients with lead colic, to whom calomel was given in sufficient quantity to affect the system.

While the discoloration of the gums is a very constant and *early* occurrence from the effects of lead, Dr. Burton is of opinion that salivation and turgidity of the gums are rare events, and not characteristic of its influence. The discoloration is also permanent—requiring a length of time to remove it—and in several instances, it continued and was manifest after death.

As this change takes place either from large doses of lead, taken internally, or its action on workers in it, Dr. Burton suggests that a knowledge of its cause may be ussefully employed in leading to an early treatment of the patient.—*Benjamin Travers, in Medico-Chirurgical Transactions,* vol. xxiii. T. R. B.
*Am. Jour. of Med. Sciences.*]

